# Microleakage of Two Bulk Fill and One Conventional Composite in Class II Restorations of Primary Posterior Teeth

**Published:** 2017-05

**Authors:** Shahram Mosharrafian, Alireza Heidari, Pegah Rahbar

**Affiliations:** 1 Assistant Professor, Dental Research Center, Dentistry Research Institute, Tehran University of Medical Sciences, Tehran, Iran; Department of Pediatric Dentistry, School of Dentistry, Tehran University of Medical Sciences, Tehran, Iran; 2 Postgraduate Student, Department of Pediatric Dentistry, School of Dentistry, Tehran University of Medical Sciences, Tehran, Iran

**Keywords:** Composite Resins, Dental Leakage, Tooth, Deciduous

## Abstract

**Objectives:**

This study aimed to assess and compare the microleakage of two bulk fill and one conventional composite in class II restorations of primary posterior teeth.

**Materials and Methods:**

This in vitro, experimental study was conducted on 60 primary mandibular second molars, which were randomly divided into three groups. Standard class II cavities were prepared in teeth and restored with 3M bulk fill composite in group 1, SonicFill bulk fill composite in group 2 and Z250 conventional composite in group 3. Single Bond 2 bonding agent was used in all cavities. The teeth were then thermocycled and immersed in 1M silver nitrate solution. The teeth were then mesiodistally sectioned and evaluated under a stereomicroscope at×10 magnification. Dye penetration depth was recorded in microns and data were analyzed using one-way ANOVA.

**Results:**

The mean (± standard deviation) dye penetration depth in the gingival margins was 543±523μm, 343±290μm and 597±590μm for 3M bulk fill, SonicFill and Z250 conventional composite, respectively. These values were 214±93μm, 302±127μm and 199±145μm in the occlusal margins, respectively. The three groups were not significantly different in terms of occlusal or gingival microleakage (P>0.05), but gingival margins showed significantly higher microleakage than occlusal margins in all three groups (P<0.05).

**Conclusions:**

Bulk fill composites are not significantly different from conventional composites in terms of microleakage.

## INTRODUCTION

Composite resins are the most commonly used direct restorative materials for restoration of dental cavities, coronal fractures, tooth wear and congenital defects of teeth due to excellent esthetic properties [1]. However, polymerization shrinkage [2–5] and its related stress [6] are among the drawbacks of composite resins. Stress due to polymerization shrinkage causes micro-cracks in composite [4] and results in debonding of material from the cavity walls and subsequent formation of micro-gaps, marginal microleakage and postoperative tooth hypersensitivity [2–5]. It is necessary to overcome the polymerization shrinkage stress of composites in order to obtain adequate marginal integrity and increase the durability of composite restorations [7, 8].

Microleakage is defined as passage of bacteria, liquids, molecules and ions through the cavity wall and restorative material, which is not clinically detectable [2, 9, 10]. It is an important factor negatively affecting the durability of restorations [2] causing tooth hyper-sensitivity, recurrent caries and pulp injury [11]. A uniform interface between the tooth and restorative material is required to seal the margins and increase the durability of restoration [12]. Obtaining such an interface is challenging for clinicians in class II composite restorations especially in the gingival margin [11]. Gingival margin is at higher risk of microleakage due to its location close to the gingival crevice [11].

Viscoelastic properties and degree of conversion of resin materials are determined by the filler content, matrix composition and polymerization mode [13, 14]. Reduction in polymerization shrinkage and subsequently decreased microleakage may be achieved by incremental application of composite resins (to decrease the C factor), use of alternative polymerization methods [15], application of a resin liner beneath the restoration [16] and increasing the filler content [17]. Although commonly used, incremental application of composite resins has drawbacks such as risk of void formation and contamination, bond failure between layers, difficult application of composite in conservative cavities and time consuming nature [18].

Attempts to decrease the microleakage and shorten the working time resulted in introduction of bulk fill composites, which have less filler content, larger filler size and higher translucency than conventional composites [19, 20]. Due to having a different monomer, bulk fill composites produce less shrinkage stress [17]. They can be applied in 4 mm thick layers without compromising their optimal mechanical properties or degree of conversion [21–24]. Lower polymerization shrinkage [25], decreased cuspal flexure in standard class II cavities [26], optimal bond strength irrespective of the cavity form and method of filling [27] and improved self-leveling ability are among other advantages of bulk fill composites [28]. Also, they are suitable for use in uncooperative patients due to faster working time [29].

SonicFill is a single-step composite that has the advantages of flowable and universal composites altogether. It has a special hand piece, which decreases the viscosity of composite upon activation of sonic energy [30].

Considering the lack of studies on application of bulk fill composites in primary teeth, this study aimed to assess the microleakage of class II composite restorations in primary posterior teeth using two bulk fill and one conventional composite.

## MATERIALS AND METHODS

This in vitro experimental study was conducted on 60 primary mandibular second molars extracted within the past six months. The teeth were sound and had no carious lesions, cracks or fracture. The study protocol was approved in the ethics committee of the School of Dentistry, Tehran University of Medical Sciences (code: IR.TUMS.VCR.REC.1395.356). Sample size was calculated to be 20 in each of the three groups according to a previous study by Poggio et al, [30] using PASS II software considering alpha=0.05, beta=0.2 and effect size of 0.42.

The teeth were stored in saline until the experiment and were then immersed in 0.5% chloramine T solution at 4°C and refrigerated for one week prior to the experiment. They were then immersed in saline again.

Two standard single box class II cavities with convergent walls and occlusogingival height of 4mm were prepared in the mesial and distal surfaces of the teeth using diamond fissure bur (No 837L/010; Tizcavan, Tehran, Iran) and high speed hand piece (Pana Max, Tokyo, Japan) under water and air spray. The buccolingual width of the cavity was 2.5mm and the isthmus width was 1.5mm.

The cervical margin of the cavity was 1mm above the cementoenamel junction. The cavity had 90° cavosurface margins.

The teeth were randomly divided into three groups (n=20) and separately mounted. A metal matrix (T-band) was applied and cavities in each group were restored by one operator (post-graduate student of pediatric dentistry) as described below. A LED light curing unit (Woodpecker, Shanghai, China) with a light intensity of 100mW/cm^2^ was used for curing in all groups and was positioned in contact with the surface. All materials were applied according to the manufacturers’ instructions.

Table 1 shows the characteristics of the materials used in this study. Group 1: The cavity was rinsed, dried and acid-etched by selective etching. Etchant was first applied on the enamel margins for five seconds and was then applied on dentin for 15 seconds, rinsed with air and water spray for 10 seconds and dried with cotton pellet. Single Bond 2 (3M ESPE, St. Paul, MN, USA) was then applied, air sprayed for 3–5 seconds from 1cm distance and cured for 20 seconds. BulkFill composite (3M ESPE, St. Paul, MN, USA) was then applied and cured for 20 seconds from the occlusal surface. Matrix band was then removed and the restoration was cured for another 20 seconds from the buccal and lingual surfaces. The restoration surface was then polished using Soflex polishing discs (3M ESPE, St. Paul, MN, USA). Group 2: Cavity preparation and etching and bonding were done as in group 1. Bulk fill composite (SonicFill, Kerr, Orange, CA, USA) was then applied using sonic hand piece and light cured from the occlusal surface for 20 seconds. Matrix band was then removed and the restoration was cured for another 20 seconds from the buccal and lingual surfaces. The restoration surface was then polished. Group 3. Cavity preparation and etching and bonding were done as in group 1.

**Table 1.** Characteristics of the materials used in this study

**Composite**	**Composition**	**Manufacturer**
Filtek Z250 (Z250, A2, N482264)	Bis-GMA, Bis-EMA, TEGDMA, UDMA zirconia, silica (82wt%, 60 vol%)	3M, ESPE, St. Paul, MN, USA
Sonicfill (SF, A2, 5026722)	Bis-GMA, TEGDMA, EBPDMA Silica, glass, oxide (83.5wt%, 69vol%)	Kerr, Orange, CA, USA
Filtek Bulkfill (FB, A2, N540884)	Bis-GMA, UDMA, Bis-EMA, procrylate resins Ytterbium trifluoride, zirconia, silica (64.5wt%, 42.5vol%)	3M, ESPE, St. Paul, MN, USA

Z250 conventional composite (3M ESPE, St. Paul, MN, USA) was incrementally applied in 2mm thickness and cured for 20 seconds from the occlusal surface. Matrix band was then removed and the restoration was cured for another 20 seconds from the buccal and lingual surfaces. The restoration surface was then polished. Working time for each composite was also recorded.

Tooth surfaces were then covered with two layers of nail varnish to 1mm around the restoration margin. Apices were sealed with wax. The teeth were then subjected to 1500 thermal cycles between 5–55°C with a dwell time of 30 seconds and transfer time of 15 seconds. Next, the teeth were immersed in water at 37°C for 24 hours and were then immersed in 1M silver nitrate solution for six hours in a dark room.

**Fig. 1:**
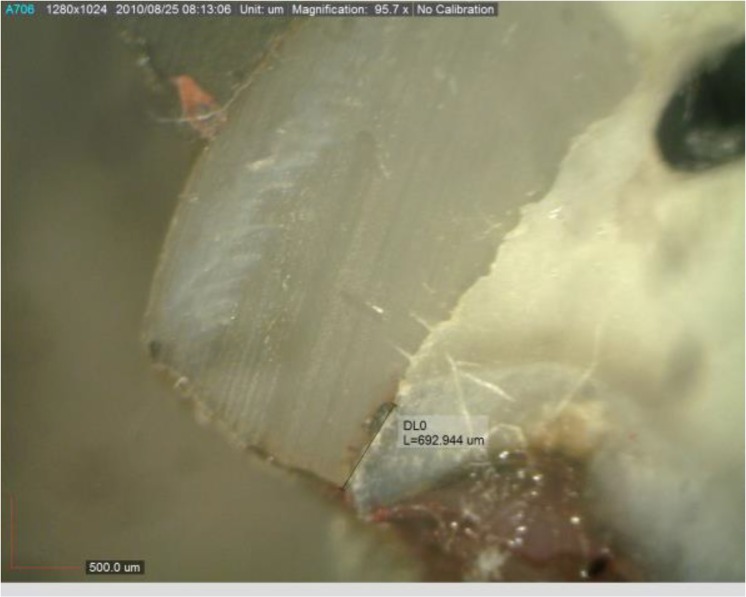
Measurement of dye penetration depth into a tooth section under a stereomicroscope at ×10 magnification

After rinsing with water, they were immersed in processing solution under fluorescent light for 12 hours. After drying, the teeth were mesiodistally sectioned by a high speed diamond saw (Mecatome T201A; Persi, Paris, France) under water coolant. Each slice was evaluated under a stereomicroscope (EZ4D Leica; Olympus, Tokyo, Japan) at×10 magnification and digitally photographed. Dye penetration depth was measured by an operator blinded to the group allocation of teeth using Las EZ version 1.6.0 software (Leica Microsystems GmbH, Wetzlar, Germany) and reported in microns (Fig. 1). Also, microleakage was determined in the occlusal and gingival margins of both boxes using the scoring system below. The highest score of each margin was recorded.

0: No dye penetration; 1: Dye penetration limited to the enamel; 2: Dye penetration extending to the external two-thirds of the gingival floor; 3: Dye penetration not reaching the axial wall; 4: Dye penetration extending to the axial wall.

Collected data were analyzed using SPSS version 21 (SPSS Inc., IL, USA). Data were not normally distributed. Thus, the microleakage score was compared among the three groups using one-way ANOVA. Wilcoxon signed rank test was used to compare the microleakage of the gingival and occlusal margins in each group.

## RESULTS

**Fig. 2:**
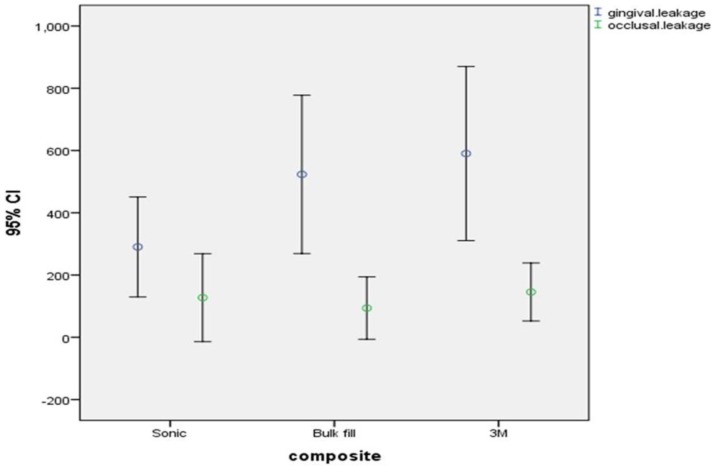
Error bar of the mean and 95% confidence interval of mean of dye penetration depth in millimeter (microleakage) for the three types of composites

No significant difference was noted among the three groups in terms of microleakage in the gingival (P=0.252) or occlusal (P=0.516) margins (Table 2). Microleakage (dye penetration depth) in the gingival margin of all groups was significantly greater than that in the occlusal margin (P=0.001 for 3M bulk fill, P=0.049 for SonicFill and P=0.001 for Z250 conventional composite). Figure 2 shows the error bar of the mean and 95% confidence interval of dye penetration depth (microleakage) for the three types of composites.

**Table 2.** Dye penetration depth in millimeters (indicative of microleakage) in the three groups

**Composite**	**Margin**	**Mean (μm)**	**Minimum (μm)**	**Maximum (μm)**	**Std. deviation**
Bulk-fill (3M)	Gingival	523.40	0.00	1508.00	543.50
	Occlusal	93.80	0.00	808.00	214.17
Bulk-fill (SonicFill)	Gingival	290.25	0.00	860.00	343.07
	Occlusal	127.35	0.00	1254.00	302.04
Conventional (Z250)	Gingival	590.20	0.00	1498.00	597.78
	Occlusal	145.45	0.00	507.00	199.14

**Fig. 3:**
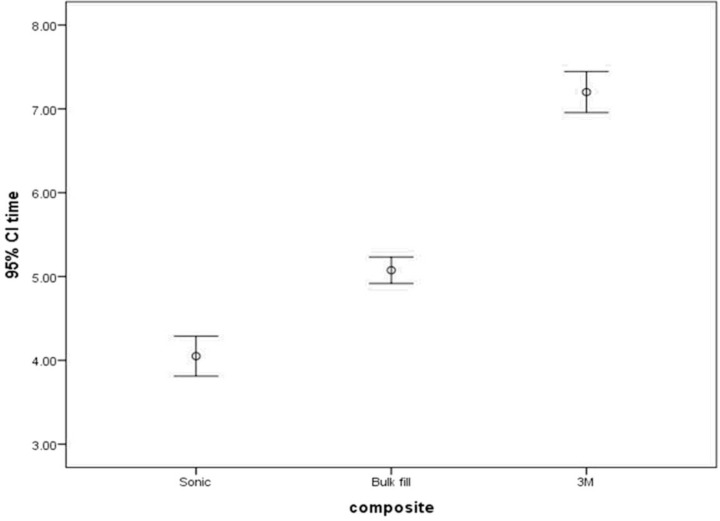
Error bar of the mean and 95% confidence interval of mean of working time in minutes with the three types of composites

The mean working time was 5.07±0.335 minutes, 4.05±0.510 minutes and 7.20±0.523 minutes in use of 3M bulk fill, SonicFill bulk fill and Z250 conventional composite, respectively. Figure 3 shows error bar of the mean and 95% confidence interval of working time with the three types of composites.

## DISCUSION

This study assessed the microleakage of class II composite restorations in primary posterior teeth using two bulk fill and one conventional composite and found no significant difference among the three groups in terms of microleakage in the gingival or occlusal margins. But in all three groups, microleakage at the gingival margin was significantly greater than that in the occlusal margin. Selection of primary mandibular second molars in this study was based on maximum standardization and elimination of the possible effect of tooth anatomy on the results. Silver nitrate was used as dye for assessment of depth of dye penetration in our study. Silver nitrate is one of the most commonly used dyes for assessment of microleakage due to better penetration ability of silver ions compared to fuchsine and methylene blue into the interface between the restorative material and tooth structure [31]. No previous study has evaluated microleakage of bulk fill composites in primary teeth. Thus, we compare our findings with those of studies on microleakage of bulk fill composites in permanent teeth. Rengo et al, [31] in 2015 evaluated marginal leakage of class II bulk fill composite restorations in permanent teeth compared to conventional composites using silver nitrate and found no significant difference between the two groups. Their findings were in line with ours although some differences in terms of bonding agent used and the evaluated margins (supragingival and subgingival margins in their study) existed between the two studies. Poggio et al, [30] evaluated microleakage of class II conventional and bulk fill composite restorations with their gingival margin below the cementoenamel junction. All composite restorations showed some degrees of microleakage in their study but SonicFill bulk fill composite showed minimum microleakage. Similarly, in our study, SonicFill showed the best results, although the difference with other groups did not reach statistical significance. This finding is attributed to the fact that SonicFill can be converted to a flowable composite for better adaptation and marginal integrity, minimizing microleakage. Eunice et al, [32] in 2012 evaluated the microleakage of SonicFill bulk fill and Filtek Supreme conventional composite restorations using technetium 99 and reported no significant difference in microleakage between the two. They also mentioned that SonicFill was faster and had easier clinical application. Their findings were in line with ours since in our study, SonicFill showed shorter working time. Leprince et al, [33] in 2014 demonstrated that bulk fill composites have lower compressive strength than nanohybrid composites but the main advantage of bulk fill composites, i.e. their fast application cannot be overlooked, which was also supported by our results. Campos et al, [34] in 2014 found no significant difference between SonicFill and conventional composites in terms of microleakage. Moorthy et al, [26] in 2012 compared cuspal flexure and microleakage of two bulk fill flowable composites and a conventional composite and showed that cuspal flexure was less in use of bulk fill flowable composites, but no significant difference was noted in cervical microleakage with the conventional composite; the latter finding was in accordance with our result. Patel et al, [35] in 2016 compared microleakage of three bulk fill and one nanohybrid composite and found no significant difference in microleakage between them but marginal microleakage in all groups was greater than occlusal microleakage; their results were in complete agreement with ours. Juloski et al, [36] in 2013 also showed greater microleakage in dentin margin of conventional and bulk fill restorations, which was in accordance with our results and may be due to the lower thickness of enamel at the gingival margin, greater distance of light curing unit from the gingival margin and weaker bond to dentin compared to enamel. Last but not least, Akah et al, [37] in a systematic review in 2016 stated that bulk fill composites provide a bond to dentin as strong as that of conventional composites without the problems related to polymerization shrinkage of conventional composites and can be very useful particularly for deep cavities.

In terms of microleakage of conventional composites, Casagrande et al, [38] compared microleakage of Filtek Z250 composite with two types of bonding agents (Scotchbond Multi-Purpose and Clearfil Mega Bond) in primary molar teeth using the same scoring system used in our study and reported score of 0 and 1 in both groups. In our study, leakage of conventional composite was up to two-thirds of the gingival floor in about 70% of the cases. The difference between our results and theirs may be due to the different dyes used (since they used methylene blue), different bonding agents used or the thermocycling protocol. In our study, leakage results in bulk fill composites were more favorable since in 85% to 90% of the cases dye penetration depth was limited to the external two-thirds of the gingival floor.

As mentioned earlier, all previous studies on bulk fill composites have been conducted on permanent teeth. Gungor et al, [39] in 2014 compared microleakage of class II restorations in primary and permanent teeth using a conventional composite and found that occlusal microleakage was not significantly different but gingival margin microleakage was greater in primary teeth, which may be due to thickness and structure of primary enamel. In general, primary enamel has less calcium and phosphorus content than permanent enamel, is thinner and has higher density of rods [40]. Primary dentin has greater diameter and number of dentinal tubules compared to permanent dentin. Thus, the available dentin substrate is less for bonding in primary teeth [41]. All these factors can affect leakage of composites in primary teeth.

Clinical studies are required to assess the clinical success of bulk fill composites in vivo. Also, future studies with the use of electron microscope are recommended for more accurate assessment of microleakage.

## CONCLUSION

Bulk fill composites have similar properties to conventional composites in terms of microleakage and may be preferred for class II restoration of primary posterior teeth to decrease working time given that their other properties are also favorable.
